# A Solvent-Free Approach to Crosslinked Hydrophobic Polymeric Coatings on Paper Using Vegetable Oil

**DOI:** 10.3390/polym14091773

**Published:** 2022-04-27

**Authors:** Amelia Loesch-Zhang, Cynthia Cordt, Andreas Geissler, Markus Biesalski

**Affiliations:** 1 Macromolecular and Paper Chemistry, Technical University Darmstadt, Alarich-Weiss-Str. 8, 64287 Darmstadt, Germany; amelia.loesch-zhang@tu-darmstadt.de (A.L.-Z.); cordt@cellulose.tu-darmstadt.de (C.C.); 2 Papiertechnische Stiftung (PTS), Pirnaer Str. 37, 01809 Heidenau, Germany

**Keywords:** vegetable oil, coatings, paper, thiol-ene, hydrophobicity, barrier, food packaging

## Abstract

Hydrophobic coatings are of utmost importance for many applications of paper-based materials. However, to date, most coating methods demand vast amounts of chemicals and solvents. Frequently, fossil-based coating materials are being used and multiple derivatization reactions are often required to obtain desired performances. In this work, we present a solvent-free paper-coating process, where olive oil as the main biogenic component is being used to obtain a hydrophobic barrier on paper. UV-induced thiol-ene photocrosslinking of olive oil was pursued in a solvent-free state at a wavelength of 254 nm without addition of photoinitiator. Optimum reaction conditions were determined in advance using oleic acid as a model compound. Paper coatings based on olive oil crosslinked by thiol-ene reaction reach water contact angles of up to 120°. By means of Fourier transform infrared spectroscopy and differential scanning calorimetry, a successful reaction and the formation of a polymer network within the coating can be proven. These results show that click-chemistry strategies can be used to achieve hydrophobic polymeric paper coatings while keeping the amount of non-biobased chemicals and reaction steps at a minimum.

## 1. Introduction

Due to the increasing importance and demand of circular and sustainable materials, paper has gained increased attention as a packaging and versatile light-weight construction material. The advantages of paper in such applications include high specific mechanical strength, the possibility to add further properties by simple coatings, the availability from renewable resources, and finally technologically well-developed recycling processes. However, with paper, some important challenges exist, such as a lack of protection against water penetration and associated changes in dimension and strength, potential damages by microorganisms, permeability to gases, absorption of odors, and sensitivity to abrasion under continuous mechanical loads. While some of these challenges can be addressed using functional additives already during paper sheet production, other aspects are tackled by subsequent finishing of readily prepared paper.

There are already several reports addressing this highly complex set of demands for paper applications. Some have focused on applying vegetable oil-based precursors onto cellulosic materials using various methods for obtaining increased hydrophobicity. For instance, cotton with low water absorbance and increased contact angle (up to 80°) was obtained via deposition of soybean oil from an organic solvent followed by thermal treatment [1]. In jute and sisal fibers coated with an emulsion of rice bran or neem oil and a phenolic resin, transesterification reactions between the triglycerides and cellulose were induced by thermal curing, resulting in increased hydrophobicity, tensile strength and durability of the fibers [2]. Chemical reactions were employed for direct covalent attachment of fatty acids to cellulosic materials via transesterification reactions using fatty acyl chlorides [3,4,5]. Ring-opening polymerization enabled crosslinking of epoxidized soybean oil onto filter paper with the cellulose hydroxyl groups acting as initiators, resulting in contact angles of up to 145° [6]. In a very interesting work, Onwukamike et al. directly grafted sunflower oil onto cellulose without any supplementary activation or derivatization steps using a 1,8-diazabicyclo[5.4.0]undec-7-ene (DBU)-CO_2_ solvent system [7]. Samyn et al. applied dispersions of different vegetable oils incorporated into poly(styrene-maleic anhydride) particles onto paper and paperboard substrates, obtaining porous coatings chemically stable against water and exhibiting static contact angles above 90° [8]. Entirely new structures can be obtained by plasma treatment, which was used by Cabrales et al. to graft oleic acid from ethanolic solution onto cotton fabric, obtaining contact angles above 150° with long-term stability [9].

Apart from covalent attachment to or physical deposition onto substrates, intensive research has recently been focused on using fatty acids or vegetable oils as sustainable resources for polymer production through crosslinking reactions [10,11,12,13,14,15,16]. Altuna et al. produced a network from epoxidized soybean oil crosslinked with an aqueous citric acid solution without the use of additional solvents or catalysts [17]. Wuzella et al. photocured acrylated epoxidized linseed oil with three different photoinitiators using a polychromatic ultraviolet (UV) lamp with a maximum light intensity at 365 nm, correlating resulting properties to cure evolution and double bond conversion [18]. Wang et al. esterified castor oil with 3-mercaptopropionic acid, which in the following allowed crosslinking of the derivatized oil films deposited on glass substrates without further addition of solvent or initiator using UV light [19].

**Figure 1:**
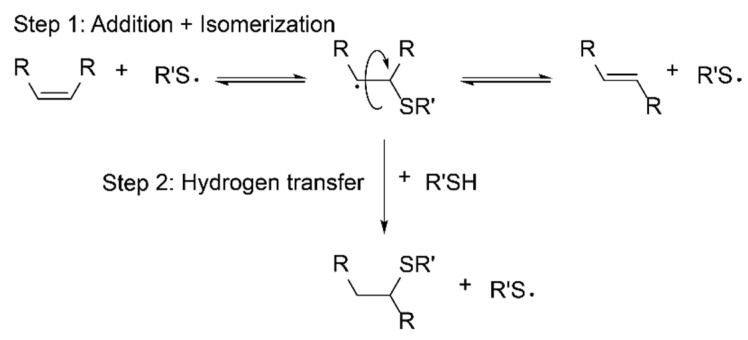
Propagation steps of the thiol-ene reaction, the first step including thiol addition via cis–trans isomerization comprising an addition–isomerization–elimination process, the second step being the rate-determining hydrogen abstraction from another thiol molecule.

To date, most of these strategies require various reaction steps, which again renders them rather sophisticated. Hence, more simple one-step procedures are increasingly in focus. An example for the latter is the well-known thiol-ene click reaction, as it can be performed on unsaturated fatty acids and vegetable oils without further derivatization. Thiol-ene reactions enjoy high popularity due to their good uniformity, controllability and insensitivity towards oxygen inhibition [20]. Thiol-ene polymerizations proceed via step-growth radical polymerization (Figure 1). The olefin structure has a decisive influence on the kinetics and conversion rates. While terminal double bonds react rapidly and quantitatively, internal enes react more slowly and without full conversion, which is due to steric effects induced by 1,3 interactions of the substituents. Internal cis-configured double bonds first isomerize to trans bonds in an insertion–isomerization–elimination process, while in the following the thiyl radical adds on the trans-configured double bond [21]. The rate-determining step of the thiol-ene-reaction is the hydrogen transfer from the thiol to the carbon-centered radical leading to product formation [22].

Due to their good controllability and simplicity, thiol-ene reactions often serve for introducing new, sometimes complex, functionalities into vegetable oils [23,24]. Several authors made use of thiol-functionalized vegetable oils to produce organic–inorganic hybrid crosslinked coatings for wood, paper and cotton textiles with (super-)hydrophobic or flame-retardant properties [25,26,27,28]. Because of the high reaction rates for terminal olefins, vegetable oils used as biobased materials for thiol-ene chemistry have frequently been functionalized with terminal double bond-containing moieties (e.g., acrylates) to allow for easier crosslinking [29,30,31]. Other research, however, has been directed towards producing crosslinked networks directly from fatty acids or vegetable oils without additional derivatization. Moser et al. synthesized linear aliphatic poly(thioether-esters) based on fatty acids by connecting C10 fatty acids and alcohols via ester chemistry followed by photoinitiator-catalyzed thiol-ene crosslinking at the unsaturated chain ends [32]. Samuelsson et al. compared reactivities and reaction rates of two different trithiols with the olefinic double bonds in methyl oleate and methyl linoleate in the presence of a photoinitiator. They found that the addition to methyl oleate occurs more quickly, which was attributed to higher cis–trans isomerization rates due to less restricted rotation, and higher reactivity of the resulting trans unsaturation compared to cis unsaturation [33]. According to Bouaziz et al., who monitored photopolymerization of olive oil with and without the presence of a thiol crosslinker, under UV irradiation thiol-ene photocrosslinking occurs much faster than oxopolymerization (under ambient air), enabling the formation of crosslinked films within only 20 s [34]. Zhao et al. produced films from linseed oil applied onto glass and metal substrates and photocrosslinked them with different thiols (both miscible and immiscible with linseed oil). Interestingly, neither photoinitiator nor solvent were required for crosslinking at irradiation wavelengths below 275 nm, with a ratio of 0.5 equivalents of thiol per double bond being sufficient for successful crosslinking [35].

The latter examples in particular show that thiol-ene chemistry has been evolved to produce networks from fatty acid or vegetable oil precursors without the use of solvents or supplementary chemicals. To date, however, the focus was mostly on the organic reactions themselves, while only in rare cases paper has been used as substrate. Coatings on paper using fatty acids or vegetable oils without the use of further derivatization or supplementary components and applying thiol-ene crosslinking reactions in solvent/catalyst-free states have yet not been reported. The latter is of great interest as by such a strategy hydrophobic surface properties can be implemented in paper with a high degree of sustainability.

Given this motivation, in this contribution, we focus on a fundamental understanding of the thiol-ene crosslinking process and its applicability with respect to biobased paper coatings. In a first step, oleic acid-based thiol-ene reactions will be examined as a model system to establish suitable crosslinking parameters. In a second part of our presented work, olive oil, containing primarily oleic acid chains, will be used to obtain polymeric hydrophobic paper coatings, and the resulting surface properties will be analyzed.

## 2. Materials and Methods

### 2.1. Materials

1,8-Octanedithiol (1,8-ODT) (99%) was purchased from Acros Organics (Geel, Belgium). Oleic acid (≥99%) was obtained from Carl Roth (Karlsruhe, Germany). 1,1,1,3,3,3-Hexamethyldisilazane (≥98%) was obtained from Merck Schuchardt (Hohenbrunn, Germany), and isopropanol and chloroform (≥99%) from Fisher Scientific (Loughborough, UK). Deuterated acetone (99.8%) was purchased from Deutero (Kastellaun, Germany). Olive oil was purchased from Oelmuehle Solling (Boffzen, Germany).

### 2.2. Methods

#### 2.2.1. Glass Wafer Hydrophobization

The glass substrate hydrophobization procedure was adapted from a procedure by Xiao et al. [36]. Glass substrates were precleaned with dish soap and distilled water followed by rinsing with isopropanol. Glass substrates were then cleaned in an ultrasonic bath by immersion into isopropanol and chloroform (7.5 min each). They were then treated by ultrasonication in a solution of 1,1,1,3,3,3-hexamethyldisilazane in chloroform (5 wt%) (15 min).

#### 2.2.2. Paper Making

Paper sheets with a grammage of 100 g/m^2^ were produced from aqueous cotton linters dispersion (2.2 wt%) using a HAAGE sheet former BBS (Haage, Peissenberg, Germany), compliant with DIN EN ISO 52692. Prior to sheet forming, the cotton linters dispersion was diluted to 0.16 wt% and stirred for 30 min to avoid entanglement of the long cotton linter fibers and obtain homogeneous paper sheets. After drying at 93 °C under reduced pressure for 10 min, grammage was determined by the bone dried paper weight. Papers were stored at norm climate conditions (23 °C, 50% relative humidity) at least for another 24 h prior to further use. This equilibration procedure is important for paper sheets to reach equilibrium water content in the sheet at the given norm climate condition.

#### 2.2.3. Coating Preparation and Crosslinking

Coatings were prepared by mixing oleic acid (10.0 g, 35.4 mmol) with 1,8-ODT (3.16 g, 17.7 mmol, 0.5 eq., corresponding to a stoichiometric C=C to SH ratio) and stirring for 30 min. Similarly, olive oil coatings were prepared from olive oil (12.0 g, 8.68 mmol) and 1,8-ODT (2.37 g, 13.2 mmol, 1.5 eq., corresponding to a stoichiometric C=C to SH ratio). Glass substrates and paper substrates used for reaction progress monitoring were coated via dip coating (home-built device) with a withdrawal speed of 2 mm/s. Paper substrates for surface analytics were impregnated with a Mathis SP 6513 size press (Mathis, Oberhasli, Switzerland) at 2 m/min speed and 0.5 bar, resulting in a final oil pick-up of (50.9 ± 0.8) wt%. UV crosslinking was performed in a Bio-Link 254 (Vilber Lourmat, Eberhardzell, Germany) at 254 nm wavelength and an irradiation dose of 20 J/cm^2^. Samples were stored overnight at ambient conditions before further analytics to ensure uniform reaction completion.

#### 2.2.4. ^1^H-NMR Spectroscopy

NMR measurements were conducted on a Bruker DRX 500 NMR spectrometer at 500 MHz, apart from measurements shown in the supporting information, which were performed on a Bruker Avance II NMR spectrometer at 300 MHz (Bruker BioSpin GmbH, Rheinstetten, Germany). Crosslinked samples were removed from the glass substrate. Samples were dissolved in deuterated acetone. Chemical shifts were calibrated to the deuterated solvent signal. Data processing was performed using MestReNova 11.0 software (Mestrelab Research S. L., Santiago de Compostela, Spain). Double bond conversions were calculated from the mean values of integrals at δ = 0.88 ppm (m, 3H, CH_3_) and δ = 5.39 ppm (m, 2H, CH=CH), integrated from three different samples. Errors were calculated from standard deviations.

#### 2.2.5. FTIR Spectroscopy

FTIR measurements were performed on Perkin Elmer Spectrum One FTIR spectrometer (PerkinElmer Instruments, Waltham, USA) in the spectral region of 650 to 4000 cm^−1^, using 4 scans with a nominal resolution of 4 cm^−1^. Spectra were recorded directly from the paper substrate or from coating material removed from the glass substrate. Data processing and background correction were performed using PerkinElmer Spectrum software.

#### 2.2.6. DSC Measurement

For DSC measurements, sample material was removed from the glass substrate and placed into 40 μL aluminum crucibles closed with a perforated lid. DSC measurements were performed using a Mettler Toledo DSC 3 apparatus (Mettler Toledo, Gießen, Germany) and data evaluation was performed using STARe software. Two heating cycles were carried out under nitrogen atmosphere in a temperature range between −75 °C and 150 °C at a heating and cooling rate of 10 K/min, with the second heating cycle being used for data evaluation.

#### 2.2.7. Contact Angle Measurement

Coated paper samples were stored at norm climate conditions for at least 24 h prior to contact angle measurement. Static contact angle measurements were performed using a Dataphysics OCA35 device (Dataphysics, Filderstadt, Germany) at standard climate conditions with 2 μL droplets of ultrapure water (Milli-Q-, Advantage A10, Millipak Express 20, (Merck Millipore, Billerica, MA, USA)). Drop shape fittings were performed using Dataphysics SCA20 software and applying the Young–Laplace fitting mode. A total of 10 measurements were performed for mean value determination for each sample, with a total of 3 samples per data point. Time-dependent contact angles were determined for a total of five measurements. Errors were calculated from the standard deviation from the mean for each sample followed by Gaussian error propagation.

#### 2.2.8. Surface Roughness Measurements

Tactile profilometry was performed on a DektakXT stylus profiler by Bruker (Bruker Nano Surfaces Business, Tucson, AZ, USA) with a 2.5 μm-diameter stylus and a stylus force of 3 mg. Measurements were performed over a z-scan range of 65.5 μm, a length of 1000 μm and a resolution of 0.111 μm/pt. A total of 10 measurements were performed per sample in two perpendicular directions to exclude any potential effects caused by sample preparation. Roughness parameters R_a_, R_q_ and R_z_ were calculated by the software according to ISO 4287 standard. Three samples were measured in total, with the mean value and the standard deviation from the mean being determined for each sample, followed by Gaussian error propagation to determine the overall error.

Optical profilometry was performed on a Sensofar PLu neox optical profiler (Sensofar-Tech, Terrassa, Spain) using a Nikon EPI 20× objective, a 850.08 × 709.35 μm scan area, a resolution of 0.69 μm/pixel, a z-scan range of 160 μm and 3 images per measurement. Surface roughness parameters S_a_, S_q_ and S_z_ were determined according to ISO 25178 standard. A total of 5 measurements were taken per sample for a total of 3 samples. The mean value and the standard deviation from the mean were determined for each sample, resulting from which the overall mean value and error, using Gaussian error propagation, were calculated.

#### 2.2.9. Optical Microscopy

Images of paper samples were obtained with a Keyence digital microscope VHX-1000 (Keyence GmbH, Neu-Isenburg, Germany) equipped with an objective VH-Z250R. Depending on the reflectivity of the specimens, a selection was made between the ring light and coaxial light modes as optimal illumination.

#### 2.2.10. SEM

For SEM measurements, samples were sputtercoated with a Pd/Pt (20/80) layer of 10 nm thickness using a Cressington Turbo 208HR sputter coater (Tescan GmbH, Dortmund, Germany). Measurements were performed on a Philips XL 30 FEG scanning electron microscope at 2 kV beam voltage, 66 μA current and 250× magnification.

## 3. Results and Discussion

In a first step, the crosslinking efficiency of dithiol linking agent 1,8-octanedithiol (1,8-ODT) for fatty acid chains was examined. Oleic acid, being the most common monounsaturated fatty acid, was chosen as a model compound, as it can at maximum form dimers when undergoing thiol-ene reactions with dithiols. This enables monitoring effects such as double bond conversion while retaining access to a higher range of analytical methods due to maintaining the solubility of the resulting compounds. The model system of oleic acid and 1,8-ODT helped to determine suitable crosslinking conditions and highlighted the significance of the thiol-component as compared to using the pure monomer at identical conditions. Secondly, the crosslinking of olive oil, a vegetable oil containing mainly oleic acid triglycerides, with 1,8-ODT was examined. The coating was applied to handsheets made from cotton linter fibers and the resulting surface morphology and water contact angles were analyzed.

1,8-ODT was chosen as thiol for its good miscibility with both oleic acid and olive oil, contrary to various commercially available tri- and tetrathiols used in preliminary experiments, while still allowing for network formation when combined with olive oil. A stoichiometric ratio of thiol functionalities to double bonds was used to ensure maximum double bond conversion. Preliminary experiments have shown that 254 nm is a suitable irradiation wavelength for crosslinking without requiring the use of curing agents or solvents, in accordance with observations made by Zhao et al. [35].

**Figure 2:**
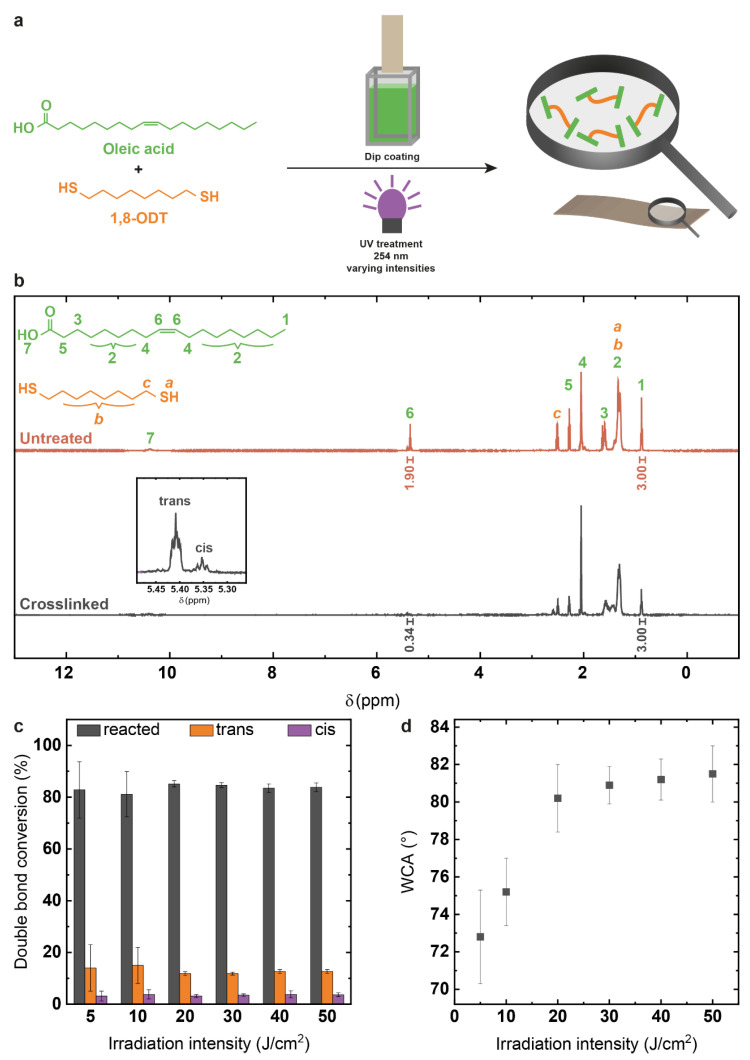
(**a**) Reaction scheme for crosslinking oleic acid with 1,8-octanedithiol and coating application; (**b**) ^1^H-NMR spectrum for untreated and crosslinked reactant mixtures as basis for calculating the double bond conversion; (**c**) double bond conversions at different irradiation intensities; (**d**) water contact angles on paper correlating to different irradiation intensities.

In the first part, a mixture of oleic acid and 1,8-ODT was applied onto both glass substrates and cotton linters paper and photocrosslinked at varying irradiation intensities (Figure 2a) in order to examine optimum reaction conditions and analyze the thiol-ene reaction process in detail.

^1^H-NMR spectroscopy (Figure 2b) allowed the quantitative examination of the double bond conversion during irradiation, as the integral associated to the two olefinic protons at 5.29–5.47 ppm decreases relative to the integral associated to the terminal methyl group at 0.80–0.92 ppm following double bond conversion. Further, the isomerization from cis double bonds (centered at 5.35 ppm), primarily present in oleic acid, to trans double bonds (centered at 5.41 ppm) can be observed as described in the literature. The latter is caused by reversible addition of thiol radicals to the double bond and results in the majority of non-reacted double bonds showing trans character [21,22,23]. ^1^H-NMR spectra of coatings deposited on glass substrates showed increasing double bond conversion with irradiation intensity, reaching a maximum of (85.2 ± 1.2)% at an irradiation intensity of 20 J/cm^2^ without significant changes at higher irradiation doses (Figure 2c), indicating that with this energy input, almost all accessible double bonds have reacted and no further crosslinking occurs at higher irradiation doses. Water contact angle (WCA) measurements of coatings deposited on cotton linters paper show similar behavior, with an augmentation of WCA values from (72.8 ± 2.5)° at 5 J/cm^2^ to (80.2 ± 1.8)° at 20 J/cm^2^ and insignificant increase at higher irradiation intensities (Figure 2d). This confirms the results obtained by ^1^H-NMR spectroscopy and indicates that the main part of the reaction including a rise of the fluid’s viscosity takes place until this point. A value of 20 J/cm^2^ was therefore selected as irradiation intensity for all future experiments.

**Figure 3:**
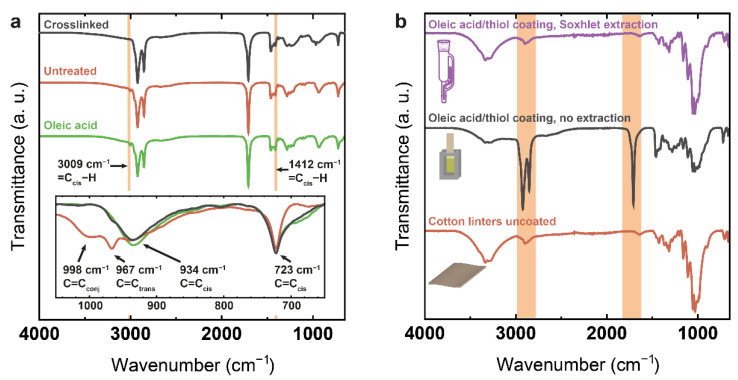
(**a**) FTIR spectra of pure oleic acid, the untreated oleic acid/thiol mixture and the crosslinked coating highlighting the transformations characterized by double bond conversion; (**b**) FTIR spectra of uncoated cotton linters paper, cotton linters paper coated with crosslinked oleic acid/thiol mixture and the same after Soxhlet extraction show that the coating is not permanently linked to substrate.

FTIR spectroscopy was used to qualitatively assess the thiol-ene reaction (Figure 3a). The bands of main interest (Table S1) are those characterizing the double bonds, in particular the C=C_cis_ stretching mode at 3009 cm^−1^, which disappears after the thiol-ene reaction, as well as the newly appearing C=C_trans_ bending mode at 967 cm^−1^ caused by cis–trans isomerization during the reaction, and the disappearing C=C_cis_ bending modes at 722 cm^−1^, 934 cm^−1^ and 1412 cm^−1^ [23,37]. A low amount of conjugated C=C bonds is also observed for the crosslinked sample at 998 cm^−1^ [33]. Theoretically, the observation of the diminishing S–H bond and forming C–S bond could also be expected, but can in reality not be observed due to its low intensity [23].

Comparison of the FTIR spectra of coated cotton linters paper before and after Soxhlet extraction shows that the coating was not covalently bound to the paper sheet. (Figure 3b). After extraction no signals are present that can be assigned to oleic acid. The latter is not unexpected as there are no double bonds in the pure cotton linters paper that would allow for direct reaction and the dimers are most likely too small to physically anchor in the paper fiber network.

The decisive relevance of the thiol as radical initiator for the oleic acid double bond conversion was also proven, as pure oleic acid coatings on glass submitted to the same irradiation conditions showed only (2.2 ± 0.5)% double bond conversion as opposed to (82.5 ± 1.2)% for the mixture. This confirms that oxidation reactions with air oxygen radicals play no significant role during the irradiation process, but may occur to a very low degree, as previously described by Zhao et al. [35]. Corresponding observations were made during WCA measurements of pure oleic acid coatings on linters paper, with a slight contact angle increase from (22.9 ± 3.0)° for untreated paper coatings to (41.3 ± 1.9)° after UV treatment, whilst a significant increase into the almost hydrophobic range at (80.2 ± 1.8)° took place after and due to UV treatment of the oleic acid/thiol mixture coating. Performing the photoinitiated thiol-ene reaction between oleic acid and 1,8-ODT allowed using a simple scaffold for determining suitable reaction conditions and ruling out significant environmental influences on the reaction.

**Figure 4:**
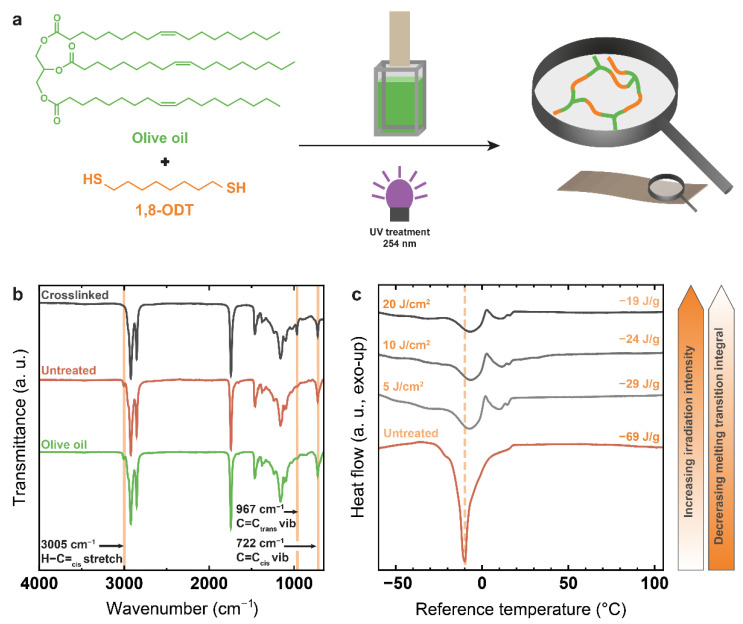
(**a**) Reaction scheme for thiol-ene reaction of olive oil with 1,8-ODT; (**b**) comparison of the FTIR spectra of fresh olive oil, the untreated olive oil/1,8-ODT mixture and the crosslinked mixture; (**c**) DSC curves of the olive oil/1,8-ODT mixture before and after crosslinking with different UV intensities showing decreasing integrals for the combined melting transitions.

The resulting knowledge was used in a second step for producing crosslinked polymer networks based on olive oil and 1,8-ODT and examining the surface properties of such coatings applied to cotton linters sheets (Figure 4a).

^1^H-NMR spectroscopy (Figure S1) revealed the olive oil batch used contained 3.03 C=C double bonds per triglyceride, as calculated from olefinic and methyl proton signal integrals, and thereby allowed calculation of the required amount of thiol to obtain a precise stoichiometric functionality ratio.

Following UV irradiation, the resulting coatings on glass substrates proved insoluble in a wide range of organic solvents, so successful network formation can be assumed. Qualitative proof of thiol-ene reaction is given by FTIR spectra (Figure 4b) of the crosslinked coating after removal from the glass substrate, as compared to pure olive oil and to the untreated olive oil/thiol mixture, showing the disappearance of the C=C band at 3005 cm^−1^ as well as trans bond formation caused by reversible thiol addition at 967 cm^−1^. DSC measurements (Figure 4c) equally point towards a successful crosslinking reaction. The thermograms show the melting peak of the untreated olive oil/thiol mixture at −10 °C prior to the crosslinking reaction. This very sharp peak represents the structurally uniform olive oil triglycerides with their rather defined melting temperature, as 1,8-ODT shows no thermal transition in the examined temperature range. After crosslinking, a strong broadening of the melting peak is observed combined with a slight shift of the maximum to −7 °C, while a second melting transition at 10–11 °C becomes visible. These changes originate from the formation of triglyceride dimers and oligomers not yet integrated into the polymeric network. The peak intensity and sum of the integrals of both melting transitions decreases with increasing irradiation intensity from −69 J/g for the untreated mixture to −19 J/g after 20 J/cm^2^ UV treatment. The decreasing integral proves that the amount of liquid low molecular weight monomer and oligomer molecules decreases. More and more olive oil molecules become integrated into the solidified crosslinked polymer network, which accordingly does not have a melting point. The main part of the reaction occurs already after submission to very low irradiation intensities below 5 J/cm^2^, as indicated by the melting transition integral, which shows the strongest decrease in that area. This corresponds to the observation made during ^1^H-NMR analysis of the oleic acid-based model system. Reference experiments of UV treatment of pure olive oil confirmed the necessity of thiol presence for successful double bond consumption reactions as equally observed in the case of oleic acid.

**Figure 5:**
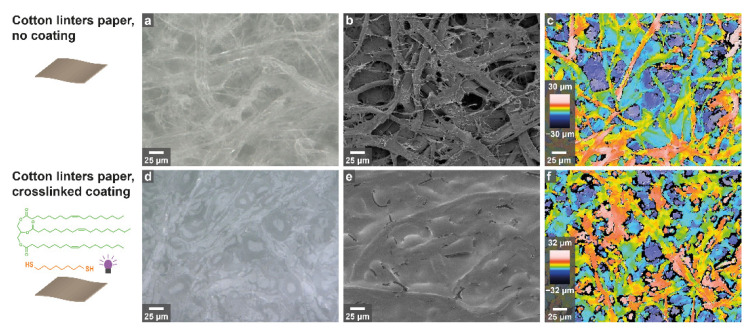
Surface characterization of uncoated (**a**–**c**) cotton linters paper and (**d**–**f**) cotton linters paper coated with olive oil/1,8-ODT using (**a**,**d**) optical microscopy, (**b**,**e**) SEM and (**c**,**f**) optical profilometry.

Following the chemical characterization, the surface properties of olive oil/1,8-ODT coatings applied onto cotton linters sheets using size pressing were examined. Optical microscopy (Figure 5a,d) and scanning electron microscopy (SEM, Figure 5b,e) allowed comparison of the sheet surface before and after coating. For uncoated cotton linters paper, individual fibers and the characteristic porous structures of the paper sheet are clearly visible and well defined both in optical microscopy and SEM, respectively. Imaging of the coated samples shows complete coverage of individual fibers with coating material, and levelling out of the entire substrate surface due to pore filling. The optical profilometry images (Figure 5c,f) show that although the fiber structure gets less distinct, the overall maxima and minima do not significantly change. Measuring roughness parameters using both tactile and optical profilometry showed that interestingly, there was no significant difference between coated and uncoated substrates, with an average roughness (R_a_) of approximately 6 μm, a root mean square roughness (R_q_) of approximately 8 μm and a z-scale roughness (R_z_) of approximately 40 μm. The surface roughness parameters (S_a_, S_q_ and S_z_) were generally slightly higher, with the most prominent difference in the z-scale area roughness that amounts to approximately 125 μm (Table 1) due to the inherent roughness of the paper surface.

**Figure 6:**
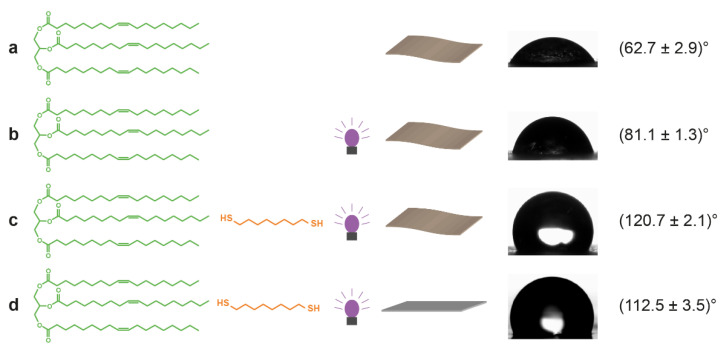
Static water contact angles (sessile drop) measured on (**a**) cotton linters paper coated with pure olive oil; (**b**) cotton linters paper coated with pure olive oil and submitted to UV irradiation; (**c**) cotton linters paper coated with the olive oil/thiol mixture and submitted to UV crosslinking; (**d**) glass coated with the olive oil/thiol mixture and submitted to UV crosslinking as a smooth reference substrate for comparison.

Static water contact angle measurements were performed in sessile drop configuration both on coated hydrophobized glass and cotton linters paper (Figure 6). Due to the high hydrophilicity and porosity of uncoated cotton linters paper, the water droplet is immediately absorbed and does not allow for contact angle investigation. Although coating with olive oil and crosslinking already lead to a significant increase in contact angles, hydrophobicity was only observed after coating with and crosslinking of the thiol-oil mixture, with the contact angle finally increasing to approximately 120°. The contact angle remained stable for at least two minutes, showing good absorption inhibition (Figure S2).

As a smooth reference substrate, coated glass showed a contact angle increase from (82.5 ± 1.1)° to (112.5 ± 3.5)°. This is significantly higher than for instance the contact angle below 90° observed for a crosslinked glass coating based on tung oil and a dithiolated isosorbide [38]. However, the superhydrophobic contact angles, approximately 160°, observed for hybrid organic–inorganic coatings are far from being attainable [25]. Covalent attachment of fatty acids or oils onto cellulosic substrates lead to similar or lower contact angles, with the exception being the metathesis polymerization resulting in contact angles of up to 145° [1,3,4,5,6].

As the surface roughness did not change significantly after coating, the increase in hydrophobicity can be attributed solely to the modification of the cellulose surface chemistry by thiol-ene photocrosslinking. These results clearly demonstrate the potential of polymer networks based on vegetable oils for use in hydrophobic paper coatings.

## 4. Conclusions and Outlook

Our report shows that it is possible to generate hydrophobic barriers on paper by applying a coating based on natural olive oil and a dithiol followed by UV-induced photocrosslinking polymerization in bulk without prior derivatization of any of the components nor the addition of a photoinitiator. The latter conclusions have been drawn from model studies using 1,8-octanedithiol as crosslinker for the natural oil. Crosslinking of the olive oil results in smooth films that exhibit contact angles well above 100° and therefore show water-repellent properties.

Avoiding the use of solvents and photoinitiators allows reducing the consumption of supplementary chemicals to an absolute minimum. Yet in our model studies, the crosslinker is not a biobased precursor. In upcoming steps, the latter will be replaced by natural, ideally low-odor or odorless, biogenic thiols. In addition, future steps will also account for investigations into material-efficient applications, barrier properties, ageing effects, recyclability and biodegradability, in order to assess a possible transfer of these model barrier films into barriers useful for packaging or construction materials.

## Figures and Tables

**Table 1:** Roughness parameters determined from tactile profilometry (profile roughness values) and optical profilometry (surface roughness values).

	R_a_ (μm)	R_q_ (μm)	R_z_ (μm)	S_a_ (μm)	S_q_ (μm)	S_z_ (μm)
Cotton linters paper, uncoated	6.3 ± 0.7	8.0 ± 0.9	39.8 ± 4.8	7.6 ± 0.6	10.0 ± 0.7	124.2 ± 12.0
Cotton linters paper, olive oil coating	6.1 ± 0.6	7.8 ± 0.8	40.8 ± 5.5	6.8 ± 0.6	9.3 ± 1.0	126.3 ± 10.8

## Data Availability

The data presented in this study are available on request from the corresponding authors.

## Supplementary Materials

The following supporting information can be downloaded at: https://www.mdpi.com/article/10.3390/polym14091773/s1, Table S1: Attribution of IR spectral bands of oleic acid/thiol mixture to functional groups; Figure S1: ^1^H-NMR spectrum of olive oil; Figure S2: Time-dependent evolution of contact angles on cotton linters paper coated with crosslinked olive oil/1,8-ODT mixture.
