# Supplementary material for: A Solvent-Free Approach to Crosslinked Hydrophobic Polymeric Coatings on Paper Using Vegetable Oil
Source: Polymers (Basel). 2022 Apr 27;14(9):1773. doi: 10.3390/polym14091773 (PMC9099761; doi:10.3390/polym14091773)
Supplement: Supplementary file 1 [file polymers-14-01773-s001.zip › polymers-1681338-supplementary.pdf]

Supporting information

A solvent-free approach to crosslinked hydrophobic polymeric coatings on paper using vegetable oil

Amelia Loesch-Zhang, Cynthia Cordt, Andreas Geissler and Markus Biesalski

Table S1: Attribution of IR spectral bands of oleic acid/thiol mixture to functional groups.[23]

**Wavenumber (cm^{-1})**	**Functional group**
3005	$H\text{-}C=_{cis}$
2957	CH_3
2924	CH_2
2852	CH_2
1709	C=O
1462	CH_2
1434	CH_3
1412	$H\text{-}C=_{cis}$
1378	CH_3
1343	O-H
1283, 1246	CH_2
1118	-C-O (ester)
1091	-C-O (ester)
998	$C=C_{conj}$*[20]
967	$C=C_{trans}$*
934	$C=C_{cis}$
723	$C=C_{cis}$ and $-(CH_2)_n-$

*only observed after crosslinking

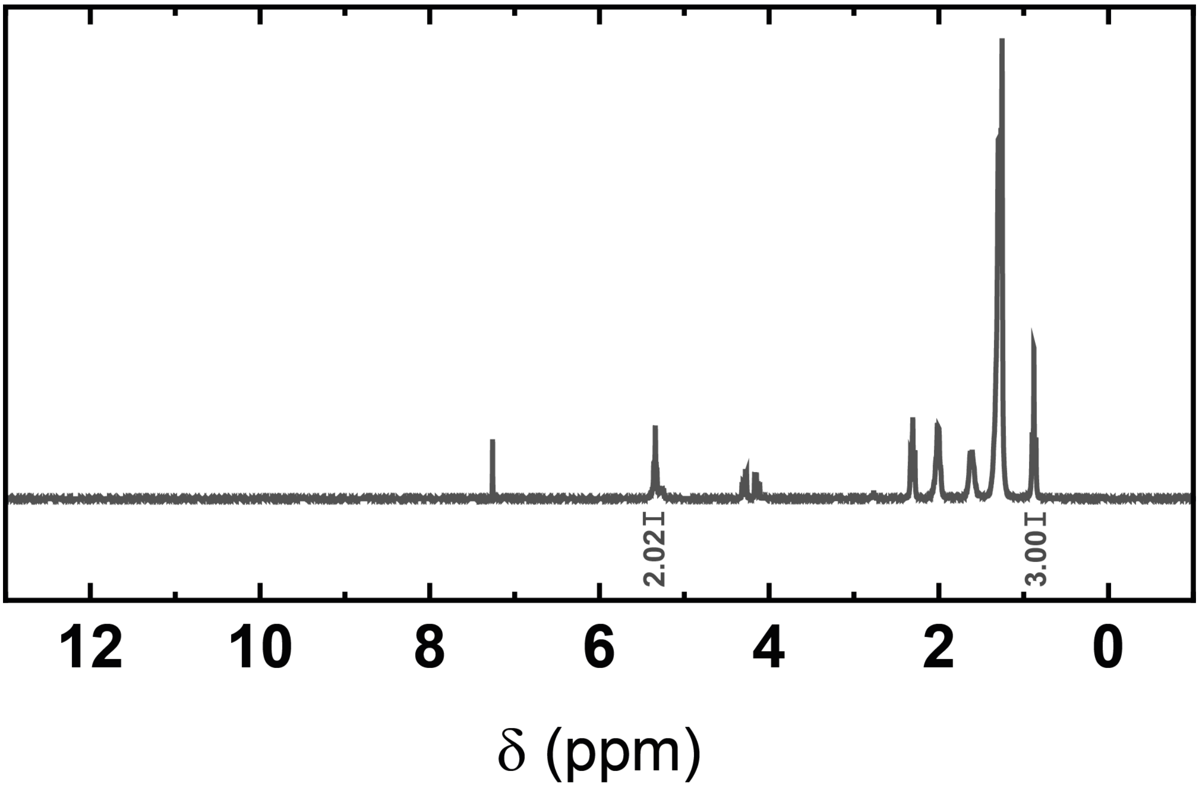

Figure S1: ^{1}H-NMR spectrum of olive oil.

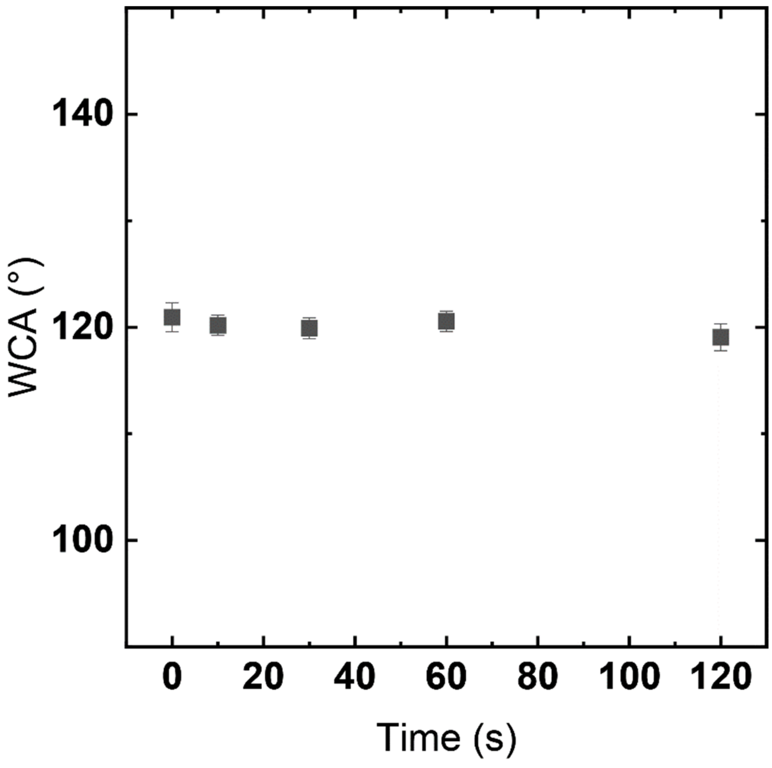

Figure S2: Time-dependent evolution of contact angles on cotton linters paper coated with crosslinked olive oil/1,8-ODT mixture.
